# The Different Systems of Medical Practice

**Published:** 1905-02

**Authors:** 


### THE DIFFERENT SYSTEMS OF MEDICAL PRACTICE.

Christian science is suggestion, plus absurdity; divine healing, suggestion, plus faith in God; Dowieism, suggestion, plus prayer and holy horror; Weltmerism, suggestion, plus imagination, pure; magnetic healing, suggestion, plus imagination also; Osteopathy, suggestion, plus massage; homeopathy, suggestion, plus nothing; allapathy, suggestion, plus tubs full of drugs, that either kill or cure; regular or rational medicine, suggestion, plus the best common horse sense available, or suggestion and medicine mixed with the best quality of brains obtainable.—*Abrams in Splanchnic Neurasthenia.*

